# Use of record linkage to evaluate treatment outcomes and trial eligibility in a real‐world metastatic prostate cancer population in Scotland

**DOI:** 10.1002/pds.4998

**Published:** 2020-04-21

**Authors:** Kelly Baillie, Tanja Mueller, Jiafeng Pan, Jennifer Laskey, Marion Bennie, Christine Crearie, Kimberley Kavanagh, Samantha Alvarez‐Madrazo, David Morrison, Julie Clarke, Aileen Keel, David Cameron, Olivia Wu, Amanj Kurdi, Robert J. Jones

**Affiliations:** ^1^ Beatson West of Scotland Cancer Centre, NHS Greater Glasgow & Clyde Glasgow UK; ^2^ Strathclyde Institute of Pharmacy and Biomedical Sciences University of Strathclyde Glasgow UK; ^3^ Department of Mathematics and Statistics University of Strathclyde Glasgow UK; ^4^ Public Health Intelligence, NHS National Services Scotland Edinburgh UK; ^5^ Institute of Health and Wellbeing University of Glasgow Glasgow UK; ^6^ Innovative Healthcare Delivery Programme (IHDP) University of Edinburgh Edinburgh UK; ^7^ Cancer Research UK Edinburgh Centre University of Edinburgh Edinburgh UK; ^8^ Institute of Cancer Sciences University of Glasgow Glasgow UK

**Keywords:** abiraterone, enzalutamide, metastatic castration‐resistant prostate cancer, pharmacoepidemiology, real world, record linkage

## Abstract

**Purpose:**

New treatments are introduced into standard care based on clinical trial results. However, it is not clear if these benefits are reflected in the broader population. This study analysed the clinical outcomes of patients with metastatic castration‐resistant prostate cancer, treated with abiraterone and enzalutamide, within the Scottish National Health Service.

**Methods:**

Retrospective cohort study using record linkage of routinely collected healthcare data (study period: February 2012 to February 2017). Overall survival (OS) was analysed using Kaplan‐Meier methods and Cox Proportional Hazard models; a subgroup analysis comprised potentially trial‐eligible patients.

**Results:**

Overall, 271 patients were included and 73.8% died during the study period. Median OS was poorer than in the pivotal trials, regardless of medication and indication: 10.8 months (95% confidence interval [CI] 8.6‐15.1) and 20.9 months (95% CI 14.9‐29.0) for abiraterone, and 12.6 months (95% CI 10.5‐18.2) and 16.0 months (95% CI 9.8—not reached) for enzalutamide, post and pre chemotherapy, respectively. Only 46% of patients were potentially “trial eligible” and in this subgroup OS improved. Factors influencing survival included baseline performance status, and baseline prostate‐specific antigen, alkaline phosphatase, and albumin levels.

**Conclusions:**

Poorer prognostic features of non‐trial eligible patients impact real‐world outcomes of cancer medicines. Electronic record linkage of routinely collected healthcare data offers an opportunity to report outcomes on cancer medicines at scale and describe population demographics. The availability of such observational data to supplement clinical trial results enables patients and clinicians to make more informed treatment decisions, and policymakers to contextualise trial findings.

**KEY POINTS**

- Median overall survival in patients with metastatic castration‐resistant prostate cancer treated with abiraterone and enzalutamide in clinical practice is less than was observed in the pivotal trials.
- Treatment outcomes were impacted by poorer prognostic factors exhibited by non‐trial eligible patients.
- The majority of patients being treated with abiraterone and enzalutamide in clinical practice would not have been eligible for inclusion in the pivotal trials that led to the approval of these medicines.
- Linked, electronic data sources can be used at a population level to describe patient demographics, estimate trial eligibility, and analyse outcomes of cancer medicines in clinical practice.
- This information, alongside clinical trial findings, may enable a more informed discussion of the likely outcomes of treatment, particularly with patients who do not fit clinical trial eligibility criteria; and facilitates a better understanding of the outcomes associated with these medicines in the “real world.”

## 1 INTRODUCTION

Cancer medicines account for a high proportion of all newly licensed medicines globally. In 2017, for example, a quarter of all medicines approved by the European Medicines Agency (EMA) and 26% of novel substances approved by the United States Food and Drug Administration (FDA) were for cancer indications.^1^, ^2^

A recent review of cancer medicines approved in Europe and the USA over the last decade found an average overall survival (OS) benefit as demonstrated in randomised clinical trials (RCTs) of 3.4 months; there were, nevertheless, wide variations across trials.^3^ Abiraterone and enzalutamide, for instance, indicated in patients with metastatic castration‐resistant prostate cancer (mCRPC) in addition to standard androgen deprivation therapy, have shown significant benefits in the pivotal trials initially when given after docetaxel‐based chemotherapy (post chemotherapy) and subsequently also in chemotherapy‐naïve patients (pre chemotherapy), with median OS ranging from 15.8 months (95% confidence interval [CI] 14.8‐17.0) in patients treated with abiraterone post chemotherapy to 35.3 months (95% CI 32.2‐not reached) in pre‐chemotherapy patients treated with enzalutamide.^4^, ^5^, ^6^, ^7^, ^8^, ^9^, ^10^

However, patient characteristics and treatment outcomes reported in a clinical trial population may not be representative of those seen in clinical practice as, by design, trial cohorts are restrictive, and participants often have fewer significant comorbidities than might be found in a broader real‐world population.^11^, ^12^ As a result, real‐world studies frequently describe considerably shorter OS than that reported from clinical trials, including studies analysing the use of abiraterone and enzalutamide,^13^, ^14^, ^15^, ^16^ although some studies have found OS rates similar to the pivotal trials.^13^, ^17^, ^18^ Consequently, a recent study perspective proposed that OS obtained from clinical trials should only be considered a surrogate endpoint for OS in the real world.^19^

As access to cancer medicines is a prominent matter of public interest and healthcare policy, policymakers—particularly in publicly funded healthcare systems such as the National Health Service (NHS) in the UK—would benefit from an improved understanding of whether these treatments deliver the same outcomes in clinical practice as demonstrated in Health Technology Assessments (HTAs), which are almost exclusively based on clinical trials data. There is a growing demand to develop more efficient methods to better identify the benefits and risks of new treatments, and thus to assure the most effective use of available resources, particularly with the increasing pressures of early licensing. Observational data have the potential to supplement the existing appraisal processes with medicines effectiveness data obtained from real‐world populations.

In 2016, funding was made available in Scotland as part of the “Beating Cancer: Ambition and Action” Cancer Plan,^20^ to deliver the Cancer Medicines Outcomes Programme (CMOP). The CMOP vision is to maximise the use of the existing and evolving local and national electronic datasets to better understand treatment outcomes of cancer medicines in the Scottish population. The methodology is designed to grow scalable and sustainable expertise in cancer medicines intelligence to drive continuous improvement in the safe and effective use of these medicines, and thereby to contribute to the international evidence base. During its initial funding phase, CMOP has sought to deliver an incremental programme of studies to test the availability and record linkage capacity of routinely collected clinical datasets within a region of Scotland.

Prostate cancer was chosen as an initial CMOP case study, as it is the most common male cancer and was the second most common cause of death in men in the United Kingdom in 2017.^21^ The West of Scotland Clinical Management Guidelines for mCRPC patients include both abiraterone and enzalutamide, and selection of treatment is made jointly by clinicians and patients taking into consideration individual patient factors and expected side effect profile.^22^ Prostate‐specific antigen (PSA) is an important molecular marker in the diagnosis and management of prostate cancer and is used alongside the Gleason score and clinical staging to evaluate the prognosis of newly diagnosed patients.^23^

The purpose of this study was to demonstrate the feasibility of using electronic record linkage (ERL) to measure real‐world outcomes of cancer medicines by determining treatment outcomes of abiraterone and enzalutamide in clinical practice in Scotland. Specific objectives were to:

- Calculate OS in patients with mCRPC treated with abiraterone and enzalutamide, and descriptively compare findings to results obtained in the respective clinical trials;
- Identify potentially trial‐eligible patients, and explore outcomes in this subgroup; and
- Analyse factors influencing survival.

## 2 METHODS

### 2.1 Study design and participants

The study was designed as a retrospective cohort study, applying ERL of routinely collected healthcare data in Scotland. The study population included all patients treated with either abiraterone or enzalutamide within NHS Greater Glasgow & Clyde (GGC). Patients were stratified as to whether or not they had previously received chemotherapy for their mCRPC in accordance with the pivotal clinical trials. NHS GGC is the largest Health Board in Scotland and provides universal access to healthcare for a population of approximately 1.2 million.

All patients who commenced treatment between February 2012 and December 2015 were identified via the Chemotherapy Electronic Prescribing and Administration System (CEPAS). CEPAS was implemented into NHS GGC systems in December 2010 and is now used for all Systemic Anti‐Cancer Therapy (SACT) prescribing within the region.^24^ Dates for cohort inclusion were chosen based on the timing of medicine approval in Scotland and data availability, allowing for sufficient follow‐up time. Patients were excluded if they participated in a clinical trial (except where the medicine was used within its product label), or if they received both abiraterone and enzalutamide during the recruitment period. Patients were followed up until death or the end of the study period (February 28, 2017), whichever occurred first.

### 2.2 Data sources

A range of data, comprising patient demographics, diagnostic details, and information regarding previous, current, and subsequent treatments, were gathered from a number of separate databases used in routine care (Table 1). Records were linked via Community Health Index (CHI) numbers, a unique patient identifier used throughout the NHS to identify individual patients.^25^

**TABLE 1:** Data sources for exposure and outcome variables used in the record linkage

Data source	Content	Purpose	Example variables
Chemotherapy Electronic Prescribing System (CEPAS)^26^	Systemic anti‐cancer therapy (SACT) prescribing for all NHS patients^a^	Identification of study population; medicine exposure	SACT medicine, dose, indication, treatment dates
Scottish Cancer Registry (SMR06)^27^	New cancer diagnoses^b^	Disease‐related details	Diagnosis (ICD‐10), date of diagnosis, Gleason score
ARIA^26^	Radiotherapy records	Previous/subsequent treatment	Dose/fraction, indication, appointment date
Scottish Care Information (SCI store)^27^	Laboratory test results	Baseline bloods; PSA levels	Type of test, test date, value
Scottish Morbidity Records, Inpatient and Day Case dataset (SMR01)^27^	Episode level data on acute hospital admissions	Comorbidities; previous/subsequent treatment	Diagnosis (ICD‐10), admission date, length of stay, procedures undertaken
Scottish Morbidity Records, Outpatient Attendance dataset (SMR00)^27^	Episode level data on outpatient clinic attendances	Comorbidities; previous/subsequent treatment	Diagnosis (ICD‐10), appointment date, procedures undertaken
Prescribing Information System (PIS)^28^	Primary care prescribing for all NHS patients^c^	Comorbidities; previous/subsequent treatment	Medicine, dose, quantity, prescription dates
National Records of Scotland (NRS)^27^	Registration of life events	Determine outcome (death)	Date and cause of death

Abbreviations: ICD‐10, International Classification of Disease, 10th edition; NHS, National Health Service; PSA, prostate‐specific antigen; SACT, systemic anti‐cancer therapy.

a CEPAS is a comprehensive source of data of all SACT prescriptions within the NHS in the West of Scotland (WoS). The numbers of patients receiving SACT within private healthcare are not known but are presumed to be very small due to the nature of the NHS (universal access, with services free at the point of delivery).

b SMR06 captures information such as TNM staging at initial diagnosis only.

c PIS does not contain information about medicines dispensed/administered in secondary care.

Trial eligibility criteria were identified through published protocols^4^, ^5^, ^6^, ^7^ and, where possible, mapped onto appropriate criteria identified in the datasets. This allowed the selection of a subset of patients likely to have fulfilled the trial selection criteria; and to subsequently compare results with those from published pivotal studies.

### 2.3 Treatment outcome measures

The primary outcome measure was OS. The duration of treatment was a key secondary endpoint. February 28, 2017, served as the censor date for those patients event free at study end.

### 2.4 Statistical analysis

Total median follow‐up was calculated in two ways. First, for descriptive purposes, using median total observation time (time from treatment initiation until death or censoring), and second, using median Kaplan‐Meier estimate of potential follow‐up to allow comparability between groups or studies with differing death rates.^29^ Median time to event, along with 95% CIs, were estimated using the Kaplan‐Meier method. Cox Proportional Hazard models were used to estimate unadjusted hazard ratios for survival, stratified by pre‐ and post‐chemotherapy medication use. To estimate the impact of prognostic variables on OS, multivariable Cox Proportional Hazard models—adjusted for Charlson comorbidity index (CCI) score and/or number of different medicines used concomitantly at baseline—were created using variables with *P* < 0.2 from the univariable analyses; the proportionality assumptions were tested using Schoenfeld residuals. In addition to the main analysis, subgroup analyses were undertaken for patients who would potentially have been eligible/non‐eligible for inclusion in the respective trials.

All analyses were performed using the R software, version 3.3.3.

## 3 RESULTS

### 3.1 Patient baseline characteristics

**FIGURE 1:**
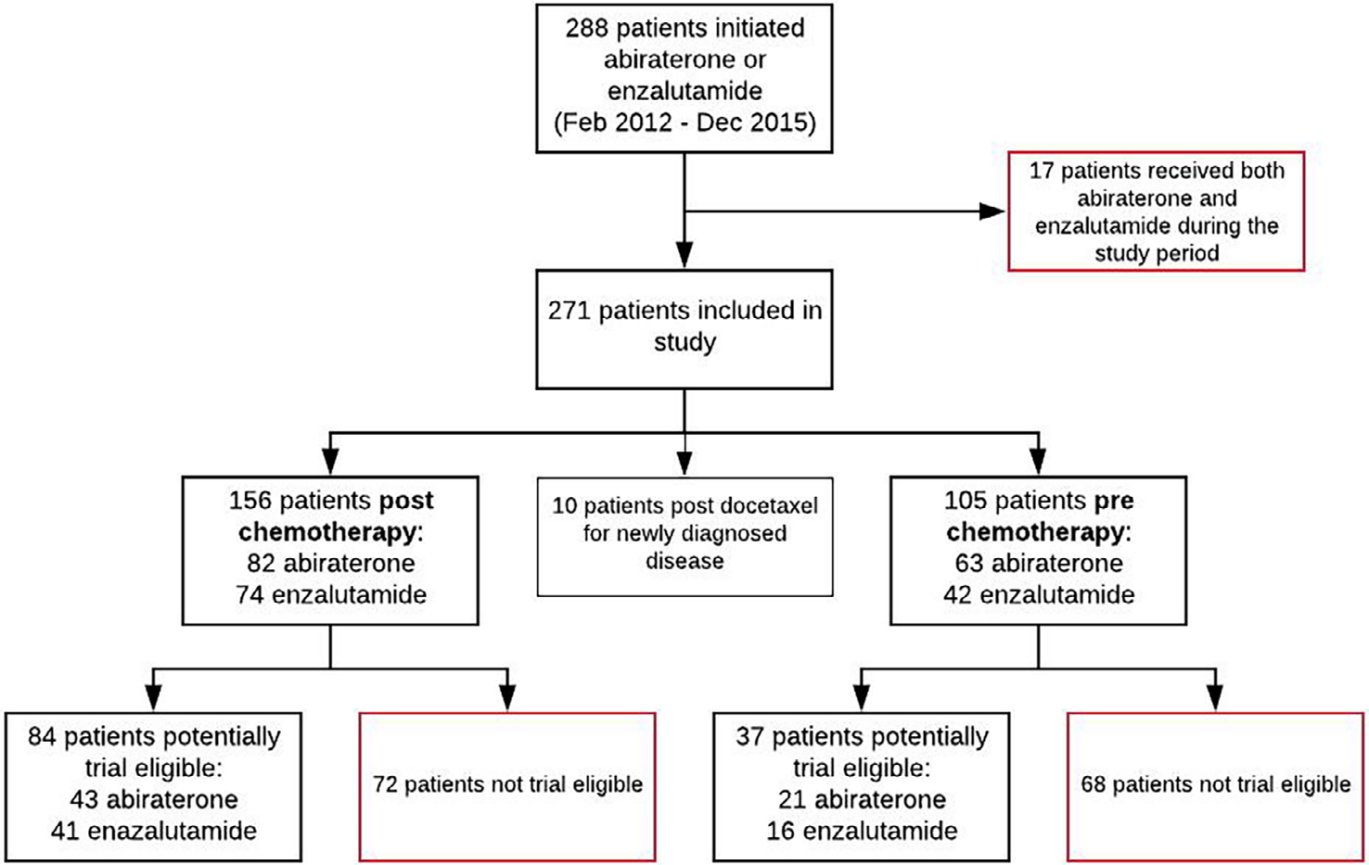
Flow chart identification of cohort population

A total of 288 patients initiated treatment with abiraterone or enzalutamide between February 2012 and December 2015. Seventeen patients were subsequently excluded due to having received both medicines during this period; hence, 271 patients were included in the analysis (Figure 1).

Baseline characteristics at treatment initiation for all patients, stratified by medicine and indication and also including those from the respective clinical trials, are summarised in Table 2.

**TABLE 2:** Study and trial patient baseline characteristics, by drug and indication

	Abiraterone				Enzalutamide			
	Post chemo	Post chemo—trial^5^	Pre chemo	Pre chemo—trial^4^	Post chemo	Post chemo—trial^6^	Pre chemo	Pre chemo—trial^7^
Number patients	82	797	63	546	74	800	42	872
Age (years)								
Median (IQR)	73 (67.3‐76.8)	69	75 (70.5‐81)	71	72 (68‐79)	69	77.5 (73‐81)	72
Range	52‐85	42‐95	58‐93	44‐95	49‐88	41‐92	63‐92	43‐93
Number patients ≥ 75 years								
N (%)	28 (34%)	220 (28%)	32 (51%)	185 (34%)	26 (35%)	199 (25%)	27 (64%)	317 (36%)
Baseline ECOG performance status								
0‐1	40 (49%)	715 (90%)	36 (57%)	546 (100%)	42 (57%)	730 (91%)	20 (48%)	872 (100%)
2‐3	6 (7%)	82 (10%)	10 (16%)	0	6 (8%)	70 (9%)	7 (17%)	0
Unavailable	36 (44%)	0	17 (27%)	0	26 (35%)	0	15 (36%)	0
Gleason score at diagnosis								
≤7	21 (26%)	341 (43%)	16 (25%)	225 (41%)	6 (8%)	360 (45%)	7 (17%)	414 (47%)
≥8	40 (49%)	356 (45%)	29 (46%)	263 (48%)	53 (72%)	366 (46%)	17 (41%)	424 (49%)
Unavailable	21 (26%)	100 (12%)	18 (29%)	58 (11%)	15 (20%)	74 (9%)	18 (43%)	34 (4%)
Only one prior chemotherapy regimen								
N (%)	79 (96%)	558 (70%)	0	0	72 (97%)	579 (72%)	0	0
Number prior docetaxel cycles								
Median (IQR)	7 (4‐10)	8	0	0	8 (4‐10)	8.5	0	0
Range	1‐13	n/a	0	0	1‐10	n/a	0	0
Baseline PSA (μg/L)								
Median (IQR)	147.9 (27.4‐430.8)	128.8	43.5 (13.8‐93.4)	42	95.9 (39‐260.6)	107.7	56.2 (15.2‐167.9)	54.1
Range	0.1‐7571	0.4‐9253	0.1‐6568	0‐3927.4	5.1‐6308	0.2‐11794	2.1‐3689	0.1‐3182
Unavailable	8 (9.8%)	9 (1%)	9 (14.3%)	0	5 (6.8%)	0	11 (26.2%)	0
Baseline haemoglobin (g/L)^a^								
Median (IQR)	116 (99‐129)	118	125 (112‐136.5)	n/a	119.5 (105‐127.8)	120	125.5 (113‐132)	130
Range	77‐164	73‐161	74‐151	n/a	67‐149	63‐156	85‐164	82‐168
Baseline alkaline phosphatase (IU/L)^a^								
Median (IQR)	171.5 (109.5‐333.8)	n/a	121 (83‐182)	93	165 (106.8‐289)	n/a	129 (93.5‐301.5)	94
Range	53‐1126	n/a	49‐2172	32‐1972	32‐3140	n/a	57‐1903	34‐4485
Baseline albumin (g/L)^a^								
Median (IQR)	33 (27‐37)	n/a	36 (32‐48)	n/a	33 (30‐36)	38	35 (32‐37.5)	38
Range	16‐42	n/a	22‐45	n/a	20‐43	26‐50	25‐41	25‐48

Abbreviations: Chemo, Chemotherapy; ECOG, Eastern Cooperative Oncology Group; IQR, interquartile range; N, number; n/a, not available; PSA, prostate specific antigen.

a Number of patients with missing data not reported due to some cells containing values <5.

### 3.2 Duration of treatment

The median duration of treatment was 9.8 months (95% CI 7.4‐11.7) for abiraterone and 7.6 months (95% CI 6.2‐10.8) for enzalutamide. Treatment duration was shorter in patients post chemotherapy, with 7.7 months (95% CI 5.7‐11.1) in the abiraterone and 7.0 months (95% CI 4.1‐10.5) in the enzalutamide group. Amongst patients treated prior to chemotherapy, the median duration of treatment was 11.1 months for both drugs (95% CI abiraterone 9.2‐14.9; 95% CI enzalutamide 6.5‐15.7). At study end, a total of 36 of the 71 patients alive (51%) remained on treatment: 20/34 (59%) on abiraterone and 16/37 (43%) on enzalutamide—however, for both medications, proportions were considerably higher in the pre‐chemotherapy groups (59% for both abiraterone and enzalutamide patients; and 50% and 29% for post‐chemotherapy abiraterone and enzalutamide, respectively).

### 3.3 Trial eligibility

A total of 70 different eligibility criteria were identified across the four trials^4^, ^5^, ^6^, ^7^:19 inclusion criteria and 51 exclusion criteria. On average, 64% of inclusion criteria (range 44%‐82% between trials) and 55% of exclusion criteria (range 33%‐74%) could be determined (Table S1).

Amongst the 261 patients being treated with abiraterone or enzalutamide either pre or post chemotherapy, an estimated 121 (46%) would have met the clinical trial eligibility criteria. Patients in the pre‐chemotherapy group were less likely to have matched the trial‐eligibility criteria (33% abiraterone, 38% enzalutamide) than in the post‐chemotherapy group (52% abiraterone, 55% enzalutamide); key ineligibilities were laboratory test results outside the required parameters at the time of screening; prior chemotherapy within a specified time frame prior to starting treatment (amongst post‐chemotherapy patients); and concomitant medication at baseline in the enzalutamide groups.

There were differences in baseline characteristics between trial eligible and ineligible patient populations, including higher baseline PSA and alkaline phosphatase levels amongst the non‐trial eligible patients; further details can be found in Tables S2 and S3.

### 3.4 Treatment outcomes

At the end of the study period, 71 patients (26%) were still alive: 34 abiraterone patients (23%) and 37 enzalutamide patients (30%); overall median OS were 14.6 months (95% CI 12.3‐17.0) and 14.0 months (95% CI 11.5‐18.2) amongst abiraterone and enzalutamide patients, respectively. Observed median OS were less than those reported from the pivotal clinical trials across all patient groups, as detailed in Tables 3 and 4.

**TABLE 3:** Outcomes in the post‐ and pre‐chemotherapy abiraterone populations

	Post chemotherapy abiraterone				Pre chemotherapy abiraterone			
Outcome Patient group	All patients	Trial eligible patients	Trial ineligible patients	Pivotal study^8^	All patients	Trial eligible patients	Trial ineligible patients	Pivotal study^9^
Number of patients	82	43	39	797	63	21	42	546
Potential follow‐up, KM estimate (months)								
Median (95% CI)	39.4 (38.9 ‐ NR)	38.9 (38.9 ‐ NR)	39.4 (NR‐NR)		16.3 (15.9‐27.1)	16.6 (15.8 ‐ NR)	16 (15.7‐ NR)	
Observed follow‐up (months)								
Median (IQR)	10.7 (4.5‐18.3)	13.9 (7.7‐22.5)	7.3 (2.6‐16.5)	20.2 (18.4‐22.1)	15.0 (11.3‐16.4)	15.9 (14.9‐20.4)	14.3 (9.4‐16.0)	49.2 (47.0‐51.8)
Mean (SD)	13.0 (10.5)				14.8 (7.6)			
Range	0.1‐45.0				0.7‐31.9			
Total	1066.8				931.2			
Patients alive at censor date^a^	(5%)	(<10%)	(<5%)	296/797 (37%)	29/63 (46%)	13/21 (62%)	16/42 (38%)	192/546 (35%)
Overall survival (95% CI) (months)	10.8 (8.6‐15.1)	13.9 (9.8‐18.3)	7.4 (4.6‐15.1)	15.8 (14.8‐17.0)	20.9 (14.9‐29)	26.7 (20.4‐NR)	14.9 (12.5‐NR)	34.7 (32.7‐36.8)

Abbreviations: CI, Confidence Interval; IQR, Inter Quartile Range; KM, Kaplan–Meier; NR, Not reached.

a Numbers of patients not reported if <5.

**TABLE 4:** Outcomes in the post‐ and pre‐chemotherapy enzalutamide populations

	Post chemotherapy enzalutamide				Pre chemotherapy enzalutamide			
Outcome Patient group	All patients	Trial eligible patients	Trial ineligible patients	Pivotal study^6^	All patients	Trial eligible patients	Trial ineligible patients	Pivotal study^10^
Number of patients	74	41	33	800	42	16	26	872
Potential follow‐up, KM estimate (months)								
Median (95% CI)	31.8 (26.5‐36.1)	27.3 (25.5‐NR)	34.2 (33.1‐NR)	14.4	20.7 (20.7‐30.3)	20.7 (19.7‐ NR)	26.9 (20.7‐NR)	31.0
Observed follow‐up (months)								
Median (IQR)	12.6 (5.3‐22.6)	16.5 (10.5‐25.1)	7.8 (2.7‐14.5)		15.6 (4.7‐20.4)	19.4 (16.5‐21.7)	9 (3.5‐17.0)	
Mean (SD)	14.6 (10.6)				14.1 (9.4)			
Range	0.4‐31.4				0.4‐31.4			
Total	1079.2				590.3			
Patients alive at censor date	18/74 (24%)	13/41 (32%)	5/33 (15%)	492/800 (62%)	17/42 (40%)	11/16 (69%)	6/26 (23%)	504/872 (58%)
Overall survival (95% CI) (months)	12.6 (10.5‐18.2)	18.2 (12.1‐30.0)	7.8 (5.2‐13.7)	18.4 (17.3‐NR)	16 (9.8‐NR)	19 (NR‐NR)	9 (4.2‐19.2)	35.3 (32.2‐NR)

Abbreviations: CI, Confidence Interval; IQR, Inter Quartile Range; KM, Kaplan–Meier; NR, Not reached.

**FIGURE 2:**
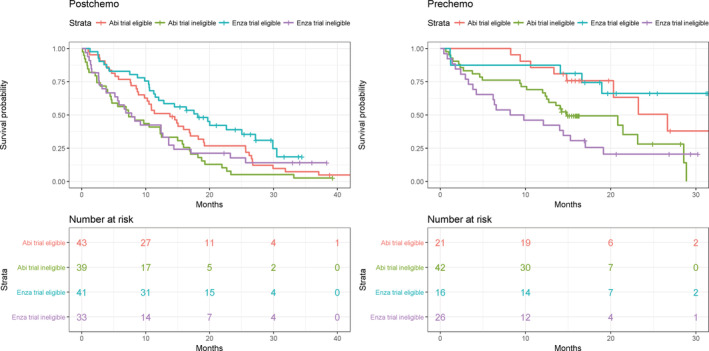
Kaplan‐Meier survival curves, by indication (post and pre chemotherapy) and stratified by medicine and trial eligibility

Results from univariable survival analyses indicated that trial eligibility significantly affected survival in both the post‐ and pre‐chemotherapy groups, with Hazard Ratios of 1.69 (95% CI 1.2‐2.37) and 2.88 (95% CI 1.55‐5.35) for potentially trial‐eligible patients vs non‐eligible patients in the post‐and pre‐chemotherapy groups, respectively. The subsequent subgroup analysis of those 121 patients who would potentially have been trial eligible found improved OS across all groups, and higher proportions of patients remained alive; at the censor date, 16 (25%) of the abiraterone and 24 (42%) of the enzalutamide patients were still alive. In particular, the OS amongst post‐chemotherapy patients was closer to the pivotal study results, with the median OS estimated at 13.9 months (95% CI 9.8‐18.3) and 18.2 months (95% CI 12.1‐30.0) for abiraterone and enzalutamide, respectively. Median OS for all patients, the potentially trial‐eligible and ineligible subgroups, and the pivotal trials are presented in Tables 3 and 4, with Kaplan‐Meier survival curves shown in Figure 2.

### 3.5 Factors influencing overall survival

Exploring the underlying prognostic characteristics which might explain differences in OS between the real‐world patients and those included in the pivotal trials showed that a variety of individual factors had an impact on survival in univariable analyses in the post‐ and/or pre‐chemotherapy group, including baseline performance status; Gleason score; and baseline PSA, alkaline phosphatase, albumin, and haemoglobin levels (see Table 5 for details).

**TABLE 5:** Survival analyses—univariable and multivariable models, by indication (post and pre chemotherapy)

	Post‐chemotherapy							Pre‐chemotherapy						
	Univariable analysis					Multivariable analysis		Univariable analysis					Multivariable analysis	
Variable	No. patients	No. person yrs follow up	No. death	Unadjusted HR (95% CI)	Global *P* value	Adjusted HR (95% CI)	*P* value	No. patients	No. person yrs follow up	No. death	Unadjusted HR (95% CI)	Global *P* value	Adjusted HR (95% CI)	*P* value
Trial eligibility^a^														
Trial eligible	84	112.8	68	1				37	56.5	13	1			
Trial ineligible	72	66.2	66	1.69 (1.20‐2.37)	.003			68	70.4	46	2.88 (1.55‐5.35)	<.001		
Medication prescribed														
Abiraterone	82	89.0	78	1	.04	1		63	77.7	34	1	.63		
Enzalutamide	74	90.0	56	0.7 (0.5‐0.99)		0.68 (0.46‐1.01)	.05	42	49.2	25	1.14 (0.68‐1.91)			
Age (years)														
≤75	104	116.8	88	1	.99			50	64.6	26	1	.21		
75+	52	62.1	46	1 (0.7‐1.43)				55	62.3	33	1.39 (0.83‐2.34)			
ECOG performance status														
0‐1	82	86.9	72	1	<.001	1		56	68.4	25	1	.10	1	
2‐3	12	4.4	12	3.62 (1.93‐6.8)		2.69 (1.36‐5.32)	.005	17	15.4	12	2.2 (1.1‐4.4)		2.46 (1.10‐5.54)	.03
<NA>	62	87.5	50	0.68 (0.47‐0.98)		0.52 (0.34‐0.81)	.004	32	43.0	22	1.37 (0.77‐2.44)		1.78 (0.85‐3.7)	.12
Charlson comorbidity index score														
0	104	128.8	84	1	.04	1		61	72.8	34	1	.93		
1	23	24.0	22	1.45 (0.9‐2.32)		0.93 (0.53‐1.64)	.80	20	25.8	11	0.9 (0.45‐1.77)			
2+	29	26.1	28	1.72 (1.11‐2.65)		1.39 (0.86‐2.24)	.18	24	28.3	14	1.03 (0.55‐1.94)			
Number of medicines prescribed concomitantly														
≤10	33	52.0	24	1	.001	1		34	49.6	14	1	.03	1	
11‐15	43	55.6	36	1.43 (0.85‐2.4)		0.74 (0.41‐1.35)	.33	33	37.8	18	1.79 (0.88‐3.63)		0.96 (0.42‐2.19)	.92
16‐20	34	34.2	32	2.11 (1.24‐3.59)		1.55 (0.83‐2.88)	.17	19	20.6	13	2.26 (1.06‐4.82)		1.37 (0.52‐3.65)	.53
21+	46	37.1	42	2.54 (1.54‐4.21)		1.60 (0.88‐2.90)	.12	19	18.9	14	2.85 (1.34‐6.03)		2.41 (0.95‐6.12)	.06
Gleason score														
≤7	35	31.6	32	1	.14	1		26	31.1	13	1	.78		
8+	85	103.2	71	0.65 (0.43‐1.00)		0.76 (0.48‐1.22)	.26	43	50.8	26	1.22 (0.63‐2.37)			
<NA>	36	44.2	31	0.67 (0.40‐1.10)		0.74 (0.42‐1.3)	.29	36	45.0	20	1.01 (0.5‐2.04)			
PSA at diagnosis^b^														
≤100	37	33.1	35	1	.09			22	23.5	11	1	.99		
101+	41	46.4	33	0.67 (0.42‐1.08)				23	27.4	13	0.98 (0.44‐2.2)			
<NA>	78	99.4	66	0.62 (0.41‐0.94)				60	76.1	35	0.96 (0.48‐1.89)			
Baseline PSA														
≤70	58	78.5	47	1	.11	1		51	69.2	21	1	.004	1	
71+	85	86.6	76	1.47 (1.02‐2.11)		1.51 (1.01‐2.25)	.04	34	34.2	27	2.64 (1.49‐4.68)		3.38 (1.70‐6.74)	.001
<NA>	13	13.9	11	1.32 (0.68‐2.54)		1.31 (0.59‐2.91)	.51	20	23.5	11	1.53 (0.74‐3.18)		2.26 (0.84‐6.04)	.10
Baseline albumin^c^														
≤34	89	81.3	78	1	.004	1		36	30.3	29	1	<.001	1	
35+	61	88.1	51	0.59 (0.42‐0.85)		0.69 (0.42‐1.11)	.12	48	67.1	21	0.32 (0.18‐0.56)		0.39 (0.20‐0.78)	.007
<NA>	6	9.6	5					21	29.4	9				
Baseline alkaline phosphatase^c^														
≤155	67	92.8	55	1	.005	1		54	71.4	27	1	.004	1	
156+	83	76.5	74	1.66 (1.16‐2.35)		1.78 (1.15‐2.75)	.01	30	26.1	23	2.34 (1.33‐4.11)		1.62 (0.82‐3.20)	.16
<NA>	6	9.6	5					21	29.4	9				
Baseline haemoglobin														
≤120	81	73.7	73	1	.007	1		34	30.8	27	1	.001	1	
121+	63	88.1	50	0.57 (0.4‐0.81)		0.99 (0.62‐1.58)	.96	47	61.5	23	0.42 (0.24‐0.73)		0.93 (0.44‐1.96)	.84
<NA>	12	17.1	11	0.64 (0.34‐1.21)		1.00 (0.36‐2.76)	.99	24	34.4	9	0.29 (0.13‐0.61)		1.46 (0.30‐7.06)	.64

Abbreviations: CI, confidence interval; ECOG, Eastern Cooperative Oncology Group; HR, hazard ratio, NA, not available; PSA, prostate specific antigen.

a Variable not included in multivariable models due to being derived from other baseline variables.

b Variable not included in multivariable models due to extent of missing data and uncertainty surrounding the accuracy of dates of diagnoses.

c Missing data not included in models due to simultaneous occurrence of absence for both albumin and alkaline phosphatase (tests always done in parallel).

In the fully adjusted multivariable analyses, factors that remained independently associated with survival were baseline performance status, and baseline PSA and alkaline phosphatase levels in the post‐chemotherapy group; and baseline performance status and baseline PSA and albumin levels in the pre‐chemotherapy group. For details, see Table 5.

In addition, a complete case analysis has been conducted to assess how sensitive the study results were to the missing data on performance status. Briefly, findings were generally in line with those obtained through the adjusted multivariable models containing missing data within the categorical variables; although some effect sizes differed slightly, the direction of effects was the same. Details are presented in Table S4.

## 4 DISCUSSION

To our knowledge, this is the first study to demonstrate the use of ERL of routinely collected data to obtain a comprehensive, population‐level assessment of treatment outcomes of cancer medicines. Furthermore, by providing results based on a subgroup analysis of potentially trial eligible patients, this study offers vital information that helps contextualise real‐world results in comparison to clinical trials.

In line with other real‐world studies, median OS in patients treated with either abiraterone or enzalutamide in both the pre and post‐chemotherapy settings were less than in the respective pivotal trials.^13^, ^14^, ^15^, ^16^ However, there were important differences between patients observed in clinical practice and the trial populations. In particular, the patients included in this study were older, and had poorer performance status; patients with performance status >2 were included within our study, but were excluded from all pivotal trials.^4^, ^5^, ^6^, ^7^ In addition, patients had higher alkaline phosphatase levels at baseline, and fewer patients had a Gleason score ≤7. Age and alkaline phosphatase were found to be significant prognostic factors in the abiraterone pre chemotherapy RCT,^9^ whilst performance status was significant in the enzalutamide post‐chemotherapy trial^6^; conversely, the post‐chemotherapy trials did not include baseline albumin,^5^, ^6^ and only the pre‐chemotherapy trials reported on baseline alkaline phosphatase values.^4^, ^7^ Comparing real‐world findings to clinical trials data is always a challenge, not least because settings, analytical methods and/or study objectives may differ considerably; hence, in order to allow truer comparisons with results based on observational studies, we would advocate for individual‐level data from trials to be made available. This would enable better replication of methods and offer deeper, more meaningful insights. Nevertheless, the prognostic significance of the patient characteristics as identified in this study also corresponds with previously published results based on observational data^11^, ^12^, ^13^^,^ 30, 31

The underlying impact of differences in patient characteristics as an explanation for the differences observed between RCTs and clinical practice has been confirmed by conducting a subgroup analysis of those patients who would potentially have been trial eligible. In this subgroup, median OS was higher than in the overall study population, indicating that real‐world outcomes are affected by an overall poorer prognosis at treatment initiation. This, in turn, raises questions regarding the appropriateness of inclusion criteria applied to RCTs—which may be overly restrictive, resulting in trial populations which could potentially diverge considerably from the patient populations subject to treatment. Clinicians and patients are thus faced with the difficult task of making treatment decisions based on extrapolating potential patient benefits in clinical practice from clinical trial cohorts.

Our findings have allowed a better understanding of the effectiveness of these medicines in real‐life clinical practice. At an individual patient level, the study results can be considered alongside clinical trial findings to enable a more informed and enriched discussion of likely treatment benefits, particularly in patients who do not fit trial eligibility criteria—for example, in patients with poorer performance status. This may be of particular benefit when medicines, including cancer treatment, adversely affect a patient's quality of life. At a population level, it is important to recognise that the effectiveness of new medicines might not be as observed in trial patient cohorts used to inform HTA models as a basis for payer/policy decision‐making. This is not to say that there is insufficient benefit (indeed our data show clear evidence of the effectiveness in the real‐world population), but it does highlight uncertainties in the magnitude of both clinical benefits and, consequently, cost‐effectiveness. Hence, the availability of rich observational data may inform a more iterative approach for assessing the value of new medicines before and after adoption into clinical practice.

### 4.1 Strengths and limitations

This study has several strengths: apart from its inclusiveness in terms of study participants, its scope with regards to the medicines studied is unprecedented (by analysing OS for both abiraterone and enzalutamide, pre and post docetaxel chemotherapy). The comprehensiveness and richness of the data available facilitated the identification of potentially trial‐eligible patients, which in turn enabled us to conduct the subgroup analysis; this provided a unique opportunity to better compare trial results to those observed in clinical practice.

Nevertheless, there are also limitations to consider. First of all, observational studies such as this are, by nature, non‐randomised and sized according to the underlying population. Consequently, there were small numbers of patients in each group, and CIs were wide and overlapped; hence, comparisons between our findings and RCT results should be interpreted with caution. Furthermore, findings are subject to confounding; however, attempts were made to adjust for confounding by using information available from electronic health records. Second, this study did not set out to compare abiraterone and enzalutamide treatments and cannot estimate the relative added benefits of these treatments since there was no control arm. In addition, it was not possible to reproduce key efficacy outcomes as reported from clinical trials; as unstructured, free text, or imaging data were not available for analyses, determining progression‐free survival, for example, was not feasible. Third, required data were not always available or were missing; for instance, 36% of patients had no performance status recorded as this only became mandatory on the chemotherapy electronic prescribing system in 2015, potentially impacting the accuracy of results. Finally, the identification of patients eligible for the pivotal clinical trials needs to be interpreted with caution; whilst certain eligibility criteria (eg, demographics and laboratory test results) were easily and unambiguously identifiable within electronic patient records, other criteria were associated with a degree of uncertainty. Identification of prior surgery, for example, was made based on assumptions regarding included procedures.

## 5 CONCLUSION

ERL of routinely collected healthcare data offers an opportunity to report outcomes on cancer medicines at scale and describe patient demographics whilst potentially identifying real‐world patients ineligible for the pivotal studies. Such information may be valuable to patients and clinicians to contextualise trial findings, and thus to make more informed shared treatment decisions.

## CONFLICT OF INTEREST

All authors declare: no support from any organisations for the submitted work; DC reports receipt of payment from Pfizer for services outside the work or the drug in question (enzalutamide). DC has been involved in a trial of this drug in breast cancer. DC has received no money personally. RJ reports he has received research funding, speaker honoraria and consultancy fees from Astellas relating to enzalutamide and from Janssen relating to abiraterone. RJ has received consultancy fees from Janssen relating to other products not related to the work. The other authors declare no competing interests.

## ETHICS STATEMENT

Ethics approval was not required. However, the use of the data use of the data was approved by the Local Privacy Advisory Committee.

## Supporting information


**Table S1.** Pivotal Clinical Trial inclusion/exclusion criteria as utilised for identification of potentially eligible patients using electronic record linkage.
**Table S2.** Baseline Characteristics of trial eligible patients.
**Table S3.** Baseline Characteristics of trial ineligible patients.
**Table S4.** Complete case analysis—multivariable survival models.
