# Supplementary material for: Use of record linkage to evaluate treatment outcomes and trial eligibility in a real‐world metastatic prostate cancer population in Scotland
Source: Pharmacoepidemiol Drug Saf. 2020 Apr 21;29(6):653–63. doi: 10.1002/pds.4998 (PMC8246935; doi:10.1002/pds.4998)
Supplement: Supplementary file 1 — Table S1. Pivotal Clinical Trial inclusion/exclusion criteria as utilised for identification of potentially eligible patients using electronic record linkage. Table S2. Baseline Characteristics of trial eligible patients. Table S3. Baseline Characteristics of trial ineligible patients. Table S4. Complete case analysis—multivariable survival models. [file PDS-29-653-s001.docx]

**Supplementary Information**

**Table S1: Pivotal Clinical Trial inclusion/exclusion criteria as utilised for identification of potentially eligible patients using electronic record linkage**

|  | **Abiraterone** | | **Enzalutamide** | |
| --- | --- | --- | --- | --- |
|  | **Post-chemotherapy** | **Pre-chemotherapy** | **Post-chemotherapy** | **Pre-chemotherapy** |
| Screening time frame | 14 days prior to 1^st^ dose | 14 days prior to 1^st^ dose | 28 days prior to 1^st^ dose | 28 days prior to 1st dose |
| Age | ≥ 18 years | ≥ 18 years | - | - |
| Previous prostate cancer treatment | 1 or 2 previous chemo regimens, one of which was docetaxel | Surgical or chemical castration; if LHRH used, treatment initiation at least 4 weeks prior to 1st dose | 1 or 2 previous chemo regimens, one of which was docetaxel; prev orchidectomy or ongoing treatment with GnRH analogues | No prior chemotherapy for prostate cancer; surgical or chemical castration |
| ECOG performance status | ≤ 2 | ≤ 1 | ≤ 2 | ≤ 2 |
| Serum testosterone | <2.0 nM | <2.0 nM | <1.2 nnml/L | <1.73 nnml/L |
| Haemoglobin | ≥ 9 g/dl | ≥ 10 g/dl | < 9 g/dL | < 9 g/dL |
| Platelet count | ≥100,000/μl | ≥100,000/μl | < 100,000/μL | < 100,000/μL |
| Serum albumin | ≥ 3.0 g/dL | ≥ 3.5 g/dL | < 30 g/L | < 30 g/L |
| Serum creatinine | < 1.5 * ULN | < 1.5 * ULN | > 177 μmol/L | > 177 μmol/L |
| Serum potassium | ≥3.5 mmol/L | ≥ 3.5 mmol/L | - | - |
| Serum bilirubin | ≥ 1.5 * ULN *[1]* | < 1.5 * ULN *[1]* | > 2 * ULN | > 2.5 * ULN |
| AST | ≥ 2.5 * ULN | < 2.5 * ULN | > 2 * ULN | > 2.5 * ULN |
| ALT | ≥ 2.5 * ULN *[2]* | < 2.5 * ULN | > 2 * ULN | > 2.5 * ULN |
| Neutrophil count | - | - | < 1500/μL | < 1500/μL |
| Comorbidities | Viral hepatitis or chronic liver disease; myocardial infarction (MI) 6 months prior; unstable angina | Viral hepatitis or chronic liver disease; MI 6 months prior to screening; unstable angina | MI within 6 months, unstable angina within 3 months; hypotension or bradycardia; TIA within 12 months; prior stroke | MI within 6 months, unstable angina within 3 months; hypotension or bradycardia; TIA within 12 months; prior stroke |
| Other prior treatment | Prostatic intervention or chemo within 30 days of first dose | - | Chemo within 4 weeks of enrolment | - |
| Prior/concomitant medication | Prior treatment with abiraterone | Opioid within 4 weeks *[3]*; itraconazole within 4 week; flutamide within 4 weeks, bicalutamide or nilutamide within 6 weeks | Prior treatment with abiraterone or enzalutamide; finasteride, dutasteride, bicalutamide, flutamide, nilutamide, or estrogens within 4 weeks; aminophylline, theophylline, bupropion, dolasetron, droperiodol, gatafloxacin, moxifloxacin, lithium, pethidine, venlafaxine, amiodarone, disopyramide, procainamide, quinidine, sotalol, maprotiline, mirtazapine within 28 days | Prior treatment with abiraterone or enzalutamide; opioid within 4 weeks *[3]*; finasteride, dutasteride, flutamide, estrogens, or cyproterone within 4 weeks; bicalutamide or nilutamide within 6 weeks |

[1] except in patients with Gilbert’s disease – assumed patients don’t have disease;

[2] except in patients with liver metastasis – assumed patients don’t have these;

[3] for cancer pain – assumed this is the indication for prescribing.

*Please note:* All inclusion criteria needed to be fulfilled in order to be eligible for the trial; exclusion from trial occurred when at least one of the exclusion criteria applied (shaded cells).

**Criteria Assumed and/or not able to be assessed within this analysis**

Due to the nature of the data available for analysis, a range of criteria originally employed in the clinical trials could not be replicated using electronic record linkage. These criteria fell broadly into three categories: first, specifics with regards to disease definitions as the basis for trial inclusion; second, comorbidities present at baseline; and third, previous treatment. The latter two categories represent exclusion criteria.

1. All four trials had specific requirements regarding ***diagnosis and disease progression*** (e.g. confirmed adenocarcinoma without neuroendocrine differentiation or small cell histology; metastatic disease confirmed by bone scan, CT, or MRI; asymptomatic or mildly symptomatic disease based on BPF-SF questionnaire). In addition, most trials required an estimated life expectancy of at least 6 months. As SMR06 does not provide this level of detail and specialist pathology/imaging records were not available, the assumption was that all patients receiving abiraterone or enzalutamide would fulfil these criteria.
2. On top of the ***comorbidities*** identified through SMR00/SMR01 records, a range of additional conditions were listed in the study protocols which would have led to trial exclusion, e.g. other malignancies, brain metastases or bone lesions; uncontrolled hypertension, history of pituitary or adrenal dysfunction, or active epidural disease; gastrointestinal disorders potentially interfering with the absorption of the drug; and possibly a range of other concurrent diseases or co-morbidities, which were not further specified but instead subsumed as “serious or uncontrolled co-existent non-malignant disease, including active and uncontrolled infection” (abiraterone) or “severe, concurrent disease, infection, or co-morbidity” (enzalutamide). In order to apply these criteria, additional details of how these comorbidities have been defined within the trials, as well as access to primary care records, would be required.
3. Previous ***treatment*** that would have led to trial exclusion included, e.g., major surgery; and radiotherapy or immunotherapy within a specified timeframe prior to treatment initiation. As with comorbidities, additional details with regards to the treatment options specified in the study protocols would be required in order to apply these criteria; however, most of this information would not have been available in the records used for analysis (e.g. ARIA records appeared to be incomplete).
4. Furthermore, limitations of the available patient records with regards to some of the criteria utilised for identification of potentially eligible patients need to be kept in mind (e.g. the identification of liver disease, heart disease, and history of seizures was not based on the complete criteria as listed in the study protocols but used a subset due to data availability).

**Table S2: Baseline Characteristics of trial eligible patients**

|  | **Abiraterone** | | **Enzalutamide** | |
| --- | --- | --- | --- | --- |
| **Characteristic** | **Post-chemo** | **Pre-chemo** | **Post-chemo** | **Pre-chemo** |
| **Number patients** | 43 | 21 | 41 | 16 |
| **Median age [years]** (IQR) | 73 (66.5 – 75.5) | 73 (72 – 81) | 72 (67 – 79) | 80.5 (74.8 – 83.3) |
| **Number patients ≥ 75 years** (%) | 12 (27.9) | 10 (47.6) | 14 (9.8) | 12 (75.0) |
| **Baseline ECOG performance status (%) ^†^** |  |  |  |  |
| 0 – 1 | 20 (46.5) | 13 (61.9) | 24 (58.5) | 9 (56.3) |
| 2 – 3 | <5 | 0 | <5 | 0 |
| **Gleason score at diagnosis (%) ^†^** |  |  |  |  |
| ≤ 7 | 10 (23.3) | 8 (38.1) | <5 | <5 |
| ≥ 8 | 21 (48.8) | 10 (47.6) | 30 (73.2) | <5 |
| **Number prior docetaxel cycles** |  |  |  |  |
| Median (IQR) | 10 (6 – 10) | 0 | 10 (7 – 10) | 0 |
| Range | 2 – 13 | 0 | 1 – 10 | 0 |
| **Baseline PSA [μg/L]** |  |  |  |  |
| Median (IQR) | 78.6  (20.1 – 227.6) | 23.3  (8.6 – 92.9) | 76.4  (49.9 – 227.2) | 15.2  (9.1 – 47.0) |
| Range | 5.0 – 1352.5 | 0.1 – 399.2 | 6.5 – 6308.0 | 4.9 – 505.4 |
| **Baseline haemoglobin [g/L]** |  |  |  |  |
| Median (IQR) | 126  (115 – 132) | 136.5  (124.8 – 141.2) | 124  (115.5 – 132) | 130.5  (120.8 – 136.5) |
| Range | 90 – 154 | 113 – 151 | 95 – 145 | 110 – 139 |
| **Baseline alkaline phosphatase [IU/L]** |  |  |  |  |
| Median (IQR) | 163.5  (106.8 – 306.8) | 110  (82.8 – 126.3) | 158.5  (107 – 246.5) | 77  (60.5 – 98) |
| Range | 53 – 1013 | 58 – 182 | 32 – 3140 | 57 – 320 |
| **Baseline albumin [g/L]** |  |  |  |  |
| Median (IQR) | 36 (33.3 – 38) | 37 (37 – 38) | 35 (33 – 36) | 35.5 (35 – 39) |
| Range | 22 – 42 | 35 – 45 | 30 – 42 | 35 – 41 |

*†-Percentages do not match up to 100% due to missing values*

*ECOG – Eastern Cooperative Oncology Group; IQR – interquartile range; PSA – prostate-specific antigen*

**Table S3: Baseline Characteristics of trial ineligible patients**

|  | **Abiraterone** | | **Enzalutamide** | |
| --- | --- | --- | --- | --- |
| **Characteristic** | **Post-chemo** | **Pre-chemo** | **Post-chemo** | **Pre-chemo** |
| **Number patients** | 39 | 42 | 33 | 26 |
| **Median age [years]** (IQR) | 72 (68.5 – 78) | 75 (68 – 81) | 73 (70 – 79) | 77 (71.3 – 79) |
| **Number patients ≥ 75 years** (%) | 16 (41.0) | 22 (52.4) | 12 (36.4) | 15 (57.7) |
| **Baseline ECOG performance status (%) ^†^** |  |  |  |  |
| 0 – 1 | 20 (51.3) | 23 (54.8) | 18 (54.5) | 11 (42.3) |
| 2 – 3 | <5 | 10 (23.8) | 5 (15.2) | 7 (26.9) |
| **Gleason score at diagnosis (%) ^†^** |  |  |  |  |
| ≤ 7 | 11 (28.2) | 8 (19.0) | <5 | <5 |
| ≥ 8 | 19 (48.7) | 19 (45.2) | 23 (69.7) | 13 (50.0) |
| **Number prior docetaxel cycles** |  |  |  |  |
| Median (IQR) | 4 (2 – 9.5) | 0 | 5 (2 – 8) | 0 |
| Range | 1 – 12 | 0 | 1 – 10 | 0 |
| **Baseline PSA [μg/L]** |  |  |  |  |
| Median (IQR) | 214.8  (91.3 – 551.2) | 50.2  (19.8 – 94.9) | 128.6  (28.8 – 312.2) | 79.4  (40.5 – 244.0) |
| Range | 0.1 – 7571.0 | 0.4 – 6567.6 | 5.1 – 1913.8 | 2.1 – 3689.3 |
| **Baseline haemoglobin [g/L]** |  |  |  |  |
| Median (IQR) | 105  (93 – 117) | 119.0  (108.5 – 130.5) | 109.5  (99.8 – 123.5) | 124.0  (112.8 – 129.0) |
| Range | 77 – 164 | 74 – 147 | 67 – 149 | 85 – 164 |
| **Baseline alkaline phosphatase [IU/L]** |  |  |  |  |
| Median (IQR) | 194.5  (111.5 – 359.2) | 153.0  (83.5 – 277.5) | 167.0  (106.2 – 305.0) | 148  (114 – 371) |
| Range | 67 – 1126 | 49 – 2172 | 54 – 1013 | 63 – 1903 |
| **Baseline albumin [g/L]** |  |  |  |  |
| Median (IQR) | 27.0 (24.3 – 31.8) | 33.0 (30.5 – 37.5) | 30.0 (26.8 – 34.0) | 34 (32 – 37) |
| Range | 16 – 41 | 22 – 42 | 20 – 43 | 25 – 41 |

*†- Percentages do not match up to 100% due to missing values*

*ECOG – Eastern Cooperative Oncology Group; IQR – interquartile range; PSA – prostate-specific antigen*

**Table S4: Complete case analysis – multivariable survival models**

|  | **Post-chemotherapy** | | | | | **Pre-chemotherapy** | | | | |
| --- | --- | --- | --- | --- | --- | --- | --- | --- | --- | --- |
| **Variable** | **No. pat.** | **Person years follow-up** | **No. deaths** | **Adjusted HR (95% CI)** | **p-value** | **No. pat.** | **Person years follow-up** | **No. deaths** | **Adjusted HR (95% CI)** | **p-value** |
| **Medication prescribed** | | | | | | | | | | |
| Abiraterone | 29 | 22.5 | 28 | 1 |  |  |  |  |  |  |
| Enzalutamide | 35 | 37.6 | 29 | 0.94  (0.47 – 1.86) | 0.86 |  |  |  |  |  |
| **ECOG performance status** | | | | | | | | | | |
| 0-1 | 57 | 58.7 | 50 | 1 |  | 40 | 45.5 | 21 | 1 |  |
| 2-3 | 7 | 1.5 | 7 | 2.78  (0.96 – 8.01) | 0.06 | 12 | 10.6 | 8 | 2.19  (0.81 – 5.96) | 0.12 |
| **Charlson comorbidity index score** | | | | | | | | | | |
| 0 | 41 | 42.7 | 35 | 1 |  |  |  |  |  |  |
| 1 | 11 | 8.1 | 11 | 0.99  (0.42 – 2.35) | 0.99 |  |  |  |  |  |
| 2+ | 12 | 9.4 | 11 | 0.74  (0.33 – 1.64) | 0.45 |  |  |  |  |  |
| **Number of medicines prescribed concomitantly** | | | | | | | | | | |
| ≤10 | 9 | 13.0 | 7 | 1 |  | 14 | 18.3 | 5 | 1 |  |
| 11-15 | 21 | 26.5 | 16 | 0.60  (0.21 – 1.69) | 0.33 | 16 | 17.2 | 8 | 1.12  (0.34 – 3.66) | 0.85 |
| 16-20 | 6 | 3.8 | 6 | 1.55  (0.44 – 5.50) | 0.49 | 8 | 6.8 | 6 | 0.93  (0.20 – 4.35) | 0.93 |
| 21+ | 28 | 16.9 | 28 | 1.90  (0.68 – 5.27) | 0.22 | 14 | 13.9 | 10 | 2.27  (0.72 – 7.14) | 0.16 |
| **Gleason score** | | | | | | | | | | |
| ≤7 | 21 | 14.5 | 21 | 1 |  |  |  |  |  |  |
| 8+ | 43 | 45.6 | 36 | 0.63  (0.31 – 1.29) | 0.20 |  |  |  |  |  |
| **Baseline PSA [μg/L]** | | | | | | | | | | |
| ≤70 | 25 | 27.4 | 21 | 1 |  | 31 | 36.1 | 13 | 1 |  |
| 71+ | 39 | 32.7 | 36 | 1.44  (0.76 – 2.70) | 0.26 | 21 | 20.0 | 16 | 2.28  (0.94 – 5.50) | 0.07 |
| **Baseline albumin [g/L]** | | | | | | | | | | |
| ≤34 | 40 | 33.5 | 36 | 1 |  | 24 | 19.8 | 18 | 1 |  |
| 35+ | 24 | 26.7 | 21 | 1.09  (0.49 – 2.39) | 0.84 | 28 | 36.3 | 11 | 0.43  (0.18 – 1.04) | 0.06 |
| **Baseline alkaline phosphatase [IU/L]** | | | | | | | | | | |
| ≤155 | 28 | 35.6 | 24 | 1 |  | 31 | 38.7 | 14 | 1 |  |
| 156+ | 36 | 24.5 | 33 | 1.73  (0.83 – 3.62) | 0.14 | 21 | 17.4 | 15 | 2.17  (0.82 – 5.77) | 0.12 |
| **Baseline haemoglobin [g/L]** | | | | | | | | | | |
| ≤120 | 39 | 27.6 | 37 | 1 |  | 23 | 21.1 | 17 | 1 |  |
| 121+ | 25 | 32.5 | 20 | 0.55  (0.22 – 1.36) | 0.19 | 29 | 35.1 | 12 | 0.99  (0.35 – 2.83) | 0.99 |

*CI – confidence interval; ECOG – Eastern Cooperative Oncology Group; HR – hazard ratio; PSA – prostate-specific antigen*
